# The Disclosure of Personally Identifiable Information in Studies of Neighborhood Contexts and Patient Outcomes

**DOI:** 10.2196/30619

**Published:** 2022-03-17

**Authors:** Andrew Graham Rundle, Michael David Miller Bader, Stephen John Mooney

**Affiliations:** 1 Department of Epidemiology Mailman School of Public Health Columbia University New York, NY United States; 2 Department of Sociology College of Arts and Sciences American University Washington, DC United States; 3 Department of Public Administration & Policy School of Public Affairs American University Washington, DC United States; 4 Department of Epidemiology School of Public Health University of Washington Seattle, WA United States

**Keywords:** geocode, patient privacy, ethical conduct of research, disclosure, privacy, security, identification, health information, strategy, outcome, neighborhood

## Abstract

Clinical epidemiology and patient-oriented health care research that incorporates neighborhood-level data is becoming increasingly common. A key step in conducting this research is converting patient address data to longitude and latitude data, a process known as geocoding. Several commonly used approaches to geocoding (eg, ggmap or the tidygeocoder R package) send patient addresses over the internet to web-based third-party geocoding services. Here, we describe how these approaches to geocoding disclose patients’ personally identifiable information (PII) and how the subsequent publication of the research findings discloses the same patients’ protected health information (PHI). We explain how these disclosures can occur and recommend strategies to maintain patient privacy when studying neighborhood effects on patient outcomes.

## Introduction

### Background

Imagine if a clinical researcher were to disclose a list of patient addresses to a third party that was outside of their hospital or health system without a formal agreement with that third party to secure the data. Imagine they then publicly announced that they disclosed the addresses, that the addresses belonged to patients with a specific disease, and that those patients were being treated at a specific hospital. The researcher’s institutional review board (IRB) and Health Insurance Portability and Accountability Act (HIPAA) compliance office would be outraged at these violations of patient privacy. Yet, this sequence of events can happen inadvertently when studying how neighborhood conditions such as access to medical facilities or surrounding food environments affect clinical outcomes in certain patient populations.

Clinical epidemiology and patient-oriented health care research incorporating neighborhood-level data is becoming progressively more common as the National Institutes of Health and professional and patient organizations are increasingly encouraging such research [1,2]. For instance, the American Cancer Society has classified multilevel research on neighborhood-level social determinants of cancer survivorship as a priority recommendation for research [2]. Here, we describe how inadvertent disclosures of personally identifiable information (PII) and protected health information (PHI) can occur when researchers study the effects of neighborhood contexts on clinical outcomes among patients and we describe ways to mitigate this risk. PII is information that can be used to identify individuals; street addresses are classified as PII [3]. PHI includes information about a patient’s past or present health conditions, any treatment for those conditions, or any other provision of care. Under HIPAA, all geographical identifiers below the state level that were created, used, or disclosed during the provision of health care are also considered PHI [4]. Patient residential addresses used during treatment, or even addresses already documented in the medical or billing records, meet the criteria for geographic PHI, as does the name of the hospital treating a patient.

**Figure 1:**
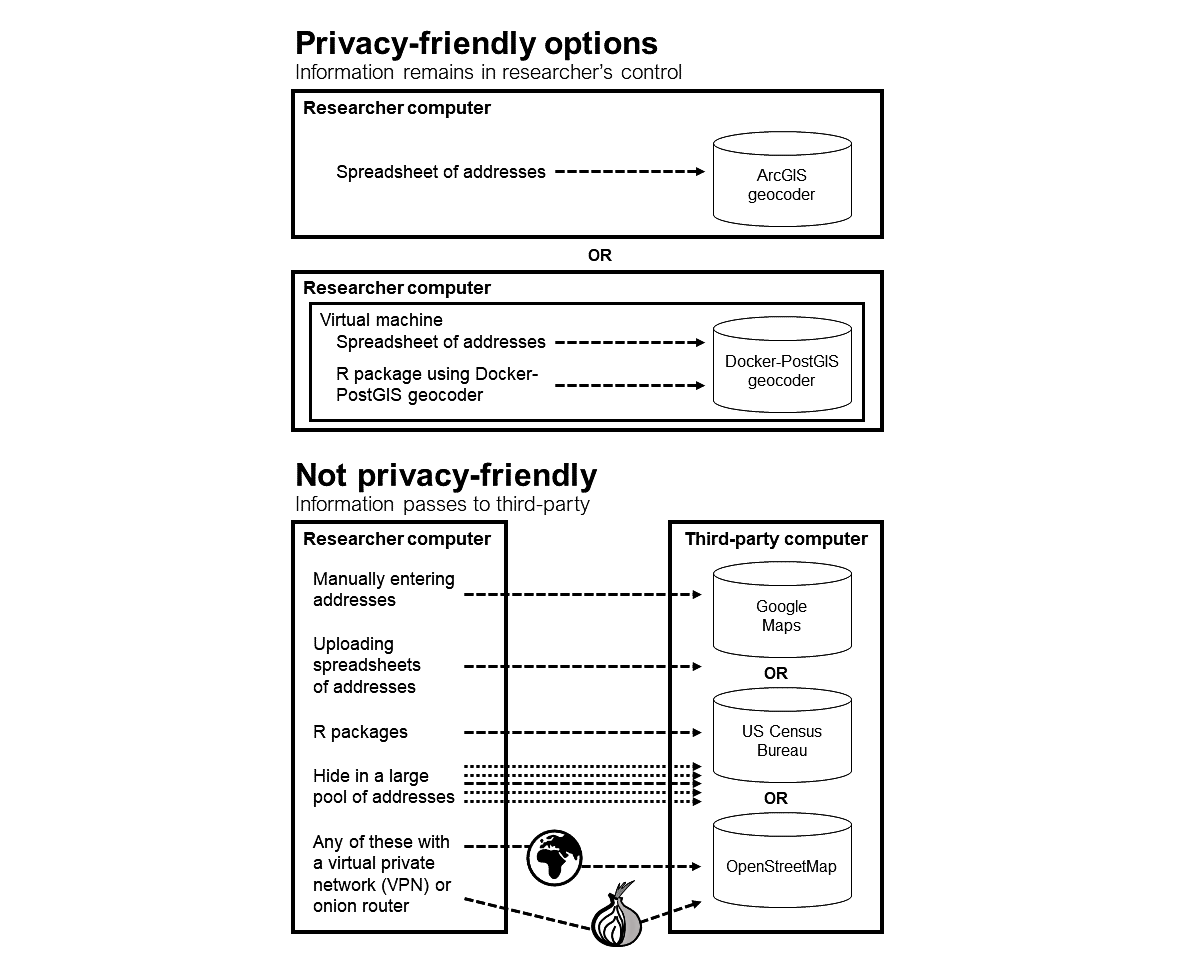
A summary of the personally identifiable information and protected health information disclosure risk for common approaches to geocoding patient addresses.

We describe the steps in the research process when PII and PHI can be disclosed when the study population is defined as patients with a specific disease or diseases, as is common in clinical studies. We then explain why many protocols do not protect patient privacy and conclude by offering suggestions for a workflow that avoids the issues we identify. We use a fictional example throughout this article to avoid compounding potential PII and PHI disclosure issues that have occurred in previously published research. The hypothetical study being referenced examines whether residential neighborhood poverty rates are associated with the risk of death among patients with COVID-19 who were treated at the Columbia University Irving Medical Center. Though fictional, the example draws on published studies and studies that we have peer reviewed for clinical and public health journals. Figure 1 summarizes the disclosure risk associated with common approaches to geocoding patient addresses.

### Privacy Pitfalls at Different Steps of Clinical Research

#### Step 1: Disclosure of Patient Addresses

The initial risk of disclosing PII and PHI occurs during a key step called geocoding. Geocoding is the process of converting a street address to longitude and latitude coordinates, which can then be linked to neighborhood-level data such as US census data. In our example of patients with COVID-19 who were treated at the Columbia University Irving Medical Center, the patients’ addresses would be geocoded as the first step in estimating poverty rates in each patient’s residential neighborhood.

Several commonly used approaches to geocoding send addresses via the internet to third-party geocoding services. These approaches include manually entering addresses into websites such as Google Maps, uploading spreadsheets of addresses to geocoding websites, and using software packages such as the R (R Foundation for Statistical Computing) packages tidygeocoder, ggmap, and googleway; the Stata command geocode; and some options included with the SAS procedure GEOCODE. The tidygeocoder R package, for example, sends addresses to geocoding web services hosted by the US Census Bureau or by OpenStreetMap (or both), and ggmap and googleway send addresses to the Google Maps geocoder [5,6]. The R package ggmap provides easy access to the web-based Google Geocoding, Distance Matrix, and Directions application programming interface (API) services and is frequently used to geocode, map, and conduct spatial analyses of patient address data via Google Maps [6]. The proliferation of easy-to-use software procedures for geocoding and spatial analysis increases the risk of inadvertent disclosures of PII. A similar situation occurs when web-based geospatial tools such as Google Street View are used to measure patient neighborhood environments [7]. Revealing PII and PHI to third parties, such as those that host geocoding tools, violates typical human subjects research protocols approved by IRBs. The first 3 examples in the bottom panel of Figure 1 show how these options are unsafe as they pass data to servers outside of the control of the researcher, to an entity not covered by a Business Associate Agreement (BAA), a contractual obligation between 2 companies to safely handle HIPAA-protected data.

#### Step 2: Disclosure of the Identity of the Health Care System

An additional layer of disclosure occurs when using web-based geocoding services because these services can link home address data that were submitted to the service to the institution from which the data originated. When researchers send geocoding requests, geocoding services capture the IP address of the computer requesting the service along with the patient home address to be geocoded. As a result, the disclosed addresses are linked to a particular health care or research institution. Furthermore, the unique API keys used for many services create a linked history of all geocoding requests submitted by a researcher. Finally, data stored in the researcher’s web browser cookies and search histories can also identify the researcher or clinician when they use some web-based geocoding services. Depending on which third-party service was used to geocode the residential addresses of the patients with COVID-19 in our example, the geocoding service provider would receive data on the identity of the institution originating the geocoding requests; they could also possibly receive data on the identity of the researcher and even information on the researcher’s recent focus on COVID-19 research. Thus, the combination of patient address data, researcher or clinician identity, and the identity of the health institution allows the third-party geocoding service to make inferences about the submitted addresses.

#### Step 3: Disclosure of Patient Health Information

Publishing research results in articles allows third parties to further contextualize the disclosed addresses. Publication itself can therefore reveal PHI that can be linked to PII [3]. Publications describing the results of research about the effects of neighborhood contexts on outcomes in patient populations typically identify the geocoding service used, the number of addresses submitted to the service, the health system the patients belong to, and the health condition(s) used as criteria for including patients in the study [8]. Publication of this study data provides sufficient information for the third party that geocoded the addresses to link patients’ individual identifiers to their health conditions and health providers [3]. For example, researchers publishing the results of the hypothetical COVID-19 study would indicate how many patients were included in the study, the method used to geocode the sample (eg, tidygeocoder and OpenStreetMap), the number of addresses successfully geocoded, and the fact that the patients received treatment for COVID-19 at the Columbia University Irving Medical Center. The combination of server logs of addresses submitted for geocoding, the capture of the IP address of the institution that originated the geocoding requests, and the information in the published paper would be sufficient for the third-party geocoding service to identify the addresses as belonging to patients with COVID-19 who had been treated at the Columbia University Irving Medical Center.

### Inadequate Strategies to Protect Patient Privacy

Some researchers recognize the risks associated with this type of study but use strategies that do not protect PII and PHI. One approach we have seen proposed is to submit patient addresses of interest to a geocoding service along with a pool of randomly selected addresses [9]. An issue with this approach is that due to referral patterns and the geographies of hospital catchment areas, patient addresses are likely to cluster and may not be sufficiently hidden by pools of randomly selected addresses. In theory, if the pool is large enough (eg, every address in the health care system’s catchment or referral area), this approach to geocoding does not provide PII to the geocoding service provider. However, this approach has several flaws. First, the approach does not follow the National Institute of Standards and Technology recommendations to secure data by requiring an encryption key rather than relying on keeping the information itself hidden [10]. Second, identifying an address list long enough to effectively obscure the patient addresses typically requires more computational skill and resources than simply geocoding the addresses securely in the first place. In our example of outcomes among patients with COVID-19, this approach might require identifying and submitting to the geocoding service every address in the Columbia University Irving Medical Center patient catchment area to fully obscure the study’s patient addresses.

Another possibility may be to use a virtual private network (VPN) that obscures the identity of the computer sending the addresses to the third-party geocoding service, thus concealing the fact that the addresses are being sent from a medical system. However, VPNs can be difficult to set up securely and many commercial VPNs do in fact reveal the location of the user’s computer [11]. Furthermore, even with a secure VPN, default settings on web browsers reveal a user’s location to the websites being visited. Thus, a researcher using a VPN to access a web-based geocoder still risks disclosing PII and PHI to the geocoder service provider. Moreover, even the successful implementation of a VPN only shifts the disclosure of the address information and researcher identity from the geocoding service provider to the VPN service provider. An onion router, such as Tor (The Onion Router), can add a layer of anonymity to ensure that the geocoding service provider cannot link the address data to a researcher, clinician, or institution [12]. However, there have been cases of onion routers being compromised [13]. The complexity of these solutions makes full compliance difficult, especially since simpler solutions exist.

### Appropriate Approaches to Geocoding Patient Data

For researchers to be compliant with HIPAA and typical IRB regulations, patient addresses should be geocoded using desktop tools, such as ArcGIS, that store address information on local secured machines. QGIS offers a free open-source alternative to ArcGIS, though default geocode options use OpenStreetMap, so care must be taken to set up desktop options. Alternatively, researchers can use a geocoder designed for server hosting within a virtual machine on their local computer. For example, the open-source PostGIS geocoder can be hosted within a Docker container and accessed using R (see [14] for an example). An alternative is for the hospital or health care system to negotiate a BAA with a third-party geocoding service [4]. Under HIPAA, a business associate provides services, including data analysis, that involve the use or disclosure of individually identifiable health information to a covered entity [4]. Although licenses for ArcGIS can be expensive (note that the various third-party services and R packages we have described are free), without a BAA, the use of third-party services to geocode patient addresses involves the inappropriate disclosure of PII and PHI. The time and administrative cost of establishing a BAA may be equivalent to, or more expensive, than an ArcGIS license.

### Conclusions

In summary, geocoding patient addresses using web-based services creates direct and large-scale disclosures of PII and PHI, and the proliferation of simple-to-use R packages for geocoding increases the risk of these inadvertent disclosures. Web-based geospatial tools are powerful and allow for efficient and rigorous research. However, researchers geocoding patient addresses must be aware of the risk of inadvertent disclosure of patient PII and PHI. Researchers should avoid web-based geocoders and use only secure desktop tools when geocoding patient information. Researchers may wish to disconnect computers from the internet while geocoding to ensure that no data are inadvertently passed to third parties.

## Abbreviations

API: application programming interface
BAA: Business Associate Agreement
HIPAA: Health Insurance Portability and Accountability Act
IRB: Institutional Review Board
VPN: virtual private network
PHI: protected health information
PII: personally identifiable information
Tor: The Onion Router
